# Community Prevalence and Predictors of Pelvic Floor-Related Symptoms in Saudi Men: Implications for Physiotherapy-Led Care

**DOI:** 10.3390/healthcare14050665

**Published:** 2026-03-06

**Authors:** Wael Alghamdi

**Affiliations:** Faculty of Nursing, Al-Baha University, Al-Baha P.O. Box 1988, Saudi Arabia; waelalghamdi@bu.edu.sa

**Keywords:** pelvic floor muscle, male pelvic health, lower urinary tract symptoms, urinary incontinence, preventive voiding behavior, smoking, physiotherapy-led care, Saudi Arabia

## Abstract

**Highlights:**

**What are the main findings?**

- In a community sample of 458 Saudi men, urinary incontinence was reported by 14% of participants, and overall pelvic floor-related symptom burden was generally low-to-moderate.
- Current smoking (OR = 1.34) and “preventive/just in case” voiding to avoid leakage (OR = 1.54) were the only significant independent predictors of higher symptom burden.

**What are the implications of the main findings?**

- Primary care screening can pragmatically target modifiable behaviors—especially smoking and preventive voiding—using brief questions to identify men at higher risk of pelvic floor-related symptoms.
- Findings support physiotherapy-led conservative pathways focusing on smoking cessation support and structured bladder training (with escalation when needed), alongside education on pelvic floor muscle training.

**Abstract:**

**Background:** Research in pelvic floor muscle-related symptoms (PFM-related symptoms) in Saudi Arabia remains limited. Clearer identification of symptom burden and its predictors is needed to guide physiotherapy strategies for prevention and management. **Objective:** We aimed to determine the prevalence of PFM-related symptoms among adult Saudi men and identify behavioral and lifestyle predictors associated with symptom burden. **Methods:** A community-based cross-sectional survey was conducted among 458 men aged >18 years from the Al-Baha region of Saudi Arabia. PFM-related symptoms were assessed using five items adapted from the Pelvic Floor Distress Inventory-20 (PFDI-20), their impact using five items from the Pelvic Floor Impact Questionnaire-7 (PFIQ-7), and urinary incontinence using the International Consultation on Incontinence Questionnaire–Urinary Incontinence Short Form (ICIQ-UI SF). Data were analyzed with descriptive statistics, correlation analysis, one-way ANOVA, and binary logistic regression. **Results:** Urinary incontinence was reported by 14% (86% reported none), but 19% disclosed regular “preventive” voiding. Symptom incidence increased with age (*p* < 0.001). Logistic regression identified smoking (OR = 1.34, *p* = 0.029) and preventive voiding (OR = 1.54, *p* = 0.002) as significant predictors of greater symptom burden. **Conclusions:** These results highlight the need for physiotherapy-led strategies in primary care, prioritizing smoking-cessation support and structured bladder training, with escalation when required. Prospective studies are needed to confirm temporality and to develop a practical rehabilitation pathway for men.

## 1. Introduction

Men experience a range of pelvic floor muscle-related symptoms (PFM-related symptoms), including urinary and bowel symptoms, sexual dysfunction, and pelvic pain; some men may experience multiple symptoms simultaneously. Pelvic floor muscles (PFMs) form a myofascial complex in the shape of a dome; they include the levator ani as well as the external urethral and anal sphincters. The muscles support the pelvic organs and regulate a number of vital functions: continence, defecation, sexual function, and lumbopelvic stability [1]. The health of these muscles can be compromised by several factors such as disuse, aging, weakness, incoordination, or hypertonicity. These factors have been reported in reviews detailing the physiology of the male pelvic floor and how to rehabilitate it when its health is impaired [2]. Pelvic therapy has been shown to produce significant health benefits, particularly in the area of sexual dysfunction and severe pelvic pain [3]. In terms of urinary health, lower urinary tract symptoms (LUTSs) are cataloged by the International Continence Society (ICS) into a range of categories: storage (urgency, frequency, nocturia), voiding (weak or interrupted stream), and post-micturition (dribble, incomplete emptying) domains; these classifications create a structure for clinical care and research [4]. Male LUTSs have an adverse impact on the quality of life of men in many countries [5]. They constitute a burden not only to individuals but also on healthcare systems and providers. However, population-based data on the prevalence and predictors of male LUTSs—particularly in Saudi Arabia—remain limited, hindering targeted prevention and physiotherapy-led care.

LUTSs are a frequent source of distress for men, particularly as they age. The EpiLUTS survey, conducted across Europe (the United Kingdom and Sweden) and the United States, reported that among men over the age of 40, 72% experienced at least one LUTS “at least sometimes”, while nearly half reported symptoms “often” [6]. The multinational EPIC study similarly documented a wide range of LUTSs, with nocturia being the most frequently reported condition (~49%); storage symptoms were more common than voiding or post-micturition symptoms [7]. These findings illustrate the progressive nature of LUTSs and their broad consequences: affected men often report a significantly impaired quality of life, both physically and psychologically [8], particularly when multiple symptoms are present simultaneously. Men with overactive bladder are also more likely to experience depression, anxiety, and reduced work performance [9,10]. Coexisting problems are frequent, with LUTSs often impairing sexual function (erectile and/or ejaculatory difficulties) and bowel health (constipation or double incontinence) [11]. Globally, LUTSs represent a major health concern, with model-based projections estimating that ~2.3 billion adults (≥20 years) had at least one LUTS by 2018, derived by applying age- and sex-specific prevalence estimates to population projections [12]. The implications for men’s health and healthcare costs are substantial. In Saudi Arabia, approximately 32% of men over the age of 40 have reported moderate-to-severe LUTSs, with the most distressing and quality-of-life-limiting symptoms being incomplete emptying and overall symptom frequency [13]. These findings highlight the importance of timely identification and effective management of LUTSs.

Treatment options for men with PFM-related symptoms range from conservative therapy and pharmacotherapy to minimally invasive procedures and surgery, all of which are recommended in current urology guidelines [14]. For non-neurogenic symptoms, international bodies, including the American Urological Association (AUA), emphasize conservative management as the primary approach. This strategy focuses on behavioral interventions—such as bladder training, timed voiding, and lifestyle modification—implemented concurrently with PFMT under the supervision of trained clinicians [15]. In post-prostatectomy cohorts, randomized trials have shown that PFMT can reduce post-micturition dribble and improve erectile function, outcomes that compare favorably with advice-only or control interventions [16]. Similarly, a multicenter trial in patients with urologic chronic pelvic pain demonstrated that pelvic-floor-directed myofascial physical therapy produced significantly better outcomes than control massage [17]. Collectively, this evidence supports rehabilitation-based management of male pelvic floor symptoms as routine practice. Central to this approach is structured patient education, which provides the foundation for physiotherapy-led conservative care.

Conservative management of non-neurogenic LUTSs typically combines behavioral strategies (e.g., bladder training, timed voiding, and fluid/caffeine management) with clinically supervised pelvic floor muscle training (PFMT) [18,19]. These approaches are recommended in current urology guidelines as first-line options in appropriate patients. Although evidence for bladder training in men is still evolving, it is commonly included within conservative care pathways [20]. In men, randomized trials suggest that targeted PFMT can reduce post-micturition dribble and may improve erectile function compared with advice-only or lifestyle guidance [21]. Additionally, a multicenter trial reported the benefits of pelvic-floor-directed myofascial physical therapy compared with control massage [17]. When synthesized, these findings advocate a pathway of rehabilitation that employs tactics of strengthening when under-activity is the problem and relaxation/coordination training when over-activity is the issue to be addressed. The crucial elements in this primary approach are the education of patients and training in the appropriate skills, together with joint decision-making, all of which create a platform for care that is led by physiotherapy.

There is a lack of community-based data on PFM-related symptoms among men in Saudi Arabia, with the limited local evidence over the past decade drawn largely from clinic-based samples [22,23]. Clinic-based studies typically capture men who seek care and may therefore over-represent more symptomatic cases, limiting generalizability to the wider community. In contrast, the present study provides community prevalence estimates and examines predictors of PFM-related symptoms among Saudi men, helping to identify potentially modifiable risk profiles relevant to physiotherapy-led prevention and conservative care. In parallel, national surveys indicate that tobacco use remains common among Saudi men (≈30% in GATS 2019) and that low physical activity is prevalent [24], highlighting opportunities for behavior-based conservative interventions. In other countries, smoking has been shown to link directly with male LUTSs, indicating that both biologically and behaviorally, it is a fruitful predictor for use in examining Saudi men [25]. Another significant observation among men with overactive bladders is that ways of coping, such as regular pre-emptive voiding, have links with higher levels of severe symptoms, which illustrates the importance of evaluating urinary experiences and ways of behaving as well as considering lifestyles [26]. Lastly, many men, including athletes, have a low awareness of PFMT and its benefits, and consequently rarely practice it [27]. Finally, awareness and practice of PFMT among men remain low in contemporary cohorts, including athletes, which supports the inclusion of PFMT awareness and skills both as predictors in epidemiologic studies and as targets for education in physiotherapy-led rehabilitation pathways.

The primary aim of this study is to identify predictors of PFM-related symptoms among Saudi men (e.g., smoking, sedentary lifestyle, age, and urinary-related behaviors). A secondary aim is to assess awareness of PFMT and the extent to which it is practiced. These objectives are intended to inform physiotherapy-led screening, referral, and education pathways.

## 2. Materials and Methods

### 2.1. Study Design and Setting

An analytical cross-sectional study on Saudi Arabian men was carried out through the framework of a self-administered Arabic questionnaire; the questions were designed to calculate the effect on sufferers of PFM-related symptoms and identify what the predictors of this were. Data collection took place in Saudi Arabia between March and August 2025. The survey link was disseminated nationally via university networks and social media platforms. STROBE guidelines were followed for the reporting of observational studies and the World Medical Association’s Declaration of Helsinki, which is under the approval of the Institutional Review Board.

### 2.2. Participants (Eligibility and Recruitment)

In order to qualify for the study, the participants had to be adult men (≥18 years) living in the Kingdom of Saudi Arabia, fluent in Arabic, and they had to give informed consent electronically. Participants who self-reported current use of diuretics or bladder stimulants were excluded. Non-probability convenience sampling was used, and the questionnaire was distributed nationally via university networks and social media platforms. This online convenience sample may under-represent older and less educated men; therefore, generalizability to the national population is limited. No identifying information was collected; however, age, sex, and region of residence were recorded. Any questionnaires that were incomplete or contained duplicated information were deleted before the analysis stage, and the final sample consisted of *n* = 458 complete responses. A single-proportion sample size calculation (95% confidence, *p* = 0.50, d = 0.05) indicated a minimum of ~385; therefore, the achieved sample (*n* = 458) exceeded this requirement.

### 2.3. Measures and Variables

The tool of this study was adapted from previous validated instruments, which were used to construct this self-administered Arabic questionnaire. The five selected PFDI items were rated on a 4-point ordinal scale: 1 = “No”, 2 = “Yes, mild discomfort”, 3 = “Yes, moderate discomfort”, and 4 = “Yes, severe discomfort”. The five-item symptom set showed high internal consistency in this sample (Cronbach’s α = 0.862). Formal construct validation (e.g., factor analysis) was not performed for this five-item symptom set; validity support was limited to expert content review and internal consistency. The questionnaire was based on Arabic versions of these instruments that have been previously translated and psychometrically evaluated in Arabic-speaking populations [28,29,30]. In addition, the selected items were reviewed by a panel of subject-matter experts to ensure clarity and relevance, supporting content validity. It includes five items from the Pelvic Floor Distress Inventory–Short Form (PFDI-20), which was used to assess PFM-related symptoms, and five items from the Pelvic Floor Impact Questionnaire–Short Form (PFIQ-7), which was used to examine their impact on daily life [31,32]. The five symptom items were selected a priori to align with the study objectives and the predefined screening-oriented outcome definition, and the final selection was confirmed through expert content review. The full PFDI-20 and PFIQ-7 were not administered to reduce respondent burden in this community survey. This selected-item set has not been previously validated as a standalone short form; therefore, content validity was supported through expert review. The International Consultation on Incontinence Questionnaire–Urinary Incontinence Short Form (ICIQ-UI SF) [30] was also employed to identify symptoms of urinary incontinence and to assess their impact on men’s daily routines; an Arabic equivalent of this form showed both validity and reliability [30,31,32]. Health and lifestyle variables—smoking habits, levels of exercise, and time spent sitting down each day—were recorded and matched with appropriate Arabic instruments used for regional demographics. Finally, social demographics including the range of ages, the participants’ marital status, where they were educated, what they did for work, and whereabouts they lived were noted.

### 2.4. Outcome Definition

The primary outcome was PFM-related symptoms. For the binary outcome, symptoms were defined as the presence of at least one of the five selected items rated ≥2 (“mild discomfort” or higher), coded as 1, versus a rating of 1 (“No”) on all five items, coded as 0. This definition was used to capture any symptom presence for community screening rather than clinically significant condition; prevalence estimates should be interpreted accordingly. An overall symptom burden score was also calculated by summing the five item scores (raw range 5–20), with higher scores indicating greater symptom burden. Urinary outcomes were derived from the Arabic ICIQ-UI Short Form (incontinence frequency, amount leaked, and impact on daily life) and analyzed as categorical predictors, alongside coping behaviors (e.g., preventive voiding) and help-seeking (e.g., physician consultation). Preventive voiding was defined as pre-emptive “just-in-case” urination (before a strong urge) and was assessed using the item “Do you try to urinate frequently as a way to prevent leakage?” (no/sometimes/yes), modeled as a categorical predictor to capture a leakage-avoidance coping behavior. The five-item PFM symptoms set showed high internal consistency in this sample, supporting aggregation to a total score. Cronbach’s α values are commonly interpreted as <0.70 poor, 0.70–0.79 acceptable, 0.80–0.89 good, and ≥0.90 excellent [33].

### 2.5. Statistical Analysis

IBM SPSS Statistics (version 27; IBM Corp., Armonk, NY, USA) were used to analyze the data. All tests were two-tailed, and *p*-value < 0.05 was considered statistically significant (α = 0.05). The primary hypotheses were that the total PFM-related symptom burden score differs across participant subgroups and that selected lifestyle/behavioral factors are associated with symptom presence (binary outcome). Accordingly, parametric tests (*t*-tests/one-way ANOVA) were used for continuous score comparisons when assumptions were met, Spearman’s ρ was used for ordinal associations, and multivariable logistic regression was used to estimate adjusted ORs for the binary outcome. Normality was assessed using the Shapiro–Wilk test and homogeneity of variances using Levene’s test; where assumptions were not met, non-parametric methods were considered. Variables were summarized as counts/percentages (categorical) and mean ± SD (scale totals). Cronbach’s α and item–total Pearson correlations were used to assess the internal consistency of the five symptom items that were derived from the PFDI. Correlation coefficients were interpreted using conventional thresholds (e.g., 0.10 small, 0.30 moderate, 0.50 large) [34]. Independent-samples *t*-tests were used for two-group comparisons of the total score. One-way ANOVA was used for univariable multi-category comparisons of the total symptom burden score (age group, marital status, education, and employment). Post hoc tests were performed if there was significance in the overall F. All candidate covariates were entered simultaneously (forced-entry method). Health conditions (multiple responses) were summarized descriptively (percentages may exceed 100%) and were not included as covariates in the primary regression model. Candidate covariates were prespecified based on the prior literature and clinical relevance, and multicollinearity was assessed using VIF and tolerance. Adjusted odds ratios (ORs) with 95% confidence intervals were reported, along with model fit indices (−2 Log Likelihood, Cox & Snell R^2^, and Nagelkerke R^2^). Goodness-of-fit (Hosmer–Lemeshow), discrimination (AUC), and multicollinearity (VIF/tolerance) were assessed for the logistic regression model. Logistic regression reflects independent associations after adjustment; bivariate findings may attenuate after multivariable adjustment. Cases with missing data on model variables were excluded from the analysis. Interaction terms were not tested due to sparse and unbalanced subgroups; future studies should assess interactions (e.g., age × smoking). Differences observed for the continuous symptom burden score (ANOVA and Spearman correlation) do not necessarily translate to the binary symptom presence outcome used in logistic regression. In addition, some sociodemographic variables may overlap with other covariates in the adjusted model, leading to attenuation of their independent effects.

### 2.6. Ethics

This study was approved by the Institutional Review Board of Al-Baha University (Approval No: 46120180/2025) approved on 23 March 2025. Informed consent was given electronically by the participants before they began to complete the questionnaire. The respondents and their data were anonymized, and the ethical principles of the study were commensurate with the Declaration of Helsinki.

## 3. Results

The PFM-related symptoms scale demonstrated strong psychometric performance, with item–total correlations varying between 0.52 and 0.75 (*p* < 0.01). Cronbach’s alpha was 0.86, which suggested a high level of internal consistency and reliability.

### 3.1. Demographic and Lifestyle Characteristics

Of the 458 respondents, the largest age group was between 35 and 55 years, accounting for 57.9%. A total of 76.4% of the men were married. Almost three-quarters (75.1%) had attained a bachelor’s degree or higher. The most prevalent type of employment was office work, representing 42.1% of the sample. Despite 28.8% reporting daily physical activity, 41.5% of participants stated that they spent between four and six hours seated each day. Additionally, 34.9% exercised several times per week, whereas 36.2% admitted that they hardly ever exercised. Approximately 50% of the respondents reported never having smoked, while 28.4% said that they were current smokers. More than half (55.3%) reported no chronic health problems; among those who did, the most common conditions were spinal or back problems (13.4%) and obesity (10.8%) (Table 1).

### 3.2. Awareness and Knowledge of PFMT Training

PFMT was known about by 63.1% of the total, with their information being gleaned mostly through social media (39.6%) and websites (29.0%). Although these information sources contained comprehensive levels of data, the respondents’ knowledge was low. As little as 13.1% of respondents knew how to practice PFMT, while just less than half of them had only a simple appreciation of it. The figures for regular practice echoed this: 11.6% did PFMT regularly, 38.9% occasionally, and 49.6% never. Although 33.7% of the respondents understood the potential advantages in terms of pain relief, and 32.9% were aware that it strengthened core muscles, 19.4% knew nothing of any benefits (Table 2).

Percentages are valid percentages; totals may not equal 100% due to rounding.

### 3.3. Urinary and PFM-Related Symptoms

Only 14% of respondents reported urinary incontinence. Of these, 9% experienced occasional leakage (about once a week), and a further 5% reported more frequent symptoms. Most leakage was described as minimal by 13.1% of respondents. In terms of causes, 7.4% attributed the leakage to urgency, while 4.6% reported that it occurred without any apparent cause. A large majority (89.1%) indicated that incontinence did not affect their daily routine, with only 3.7% reporting a moderate or severe impact. Coping methods were limited: 19% reported urinating frequently to avoid leakage, but fewer than 5% had ever sought medical help; in total, 95.4% had never consulted a healthcare professional.

The most commonly reported specific PFM-related symptom was trouble or discomfort during defecation (mean = 1.40). This was followed by incomplete bladder emptying (mean = 1.36), heaviness or pressure in the pelvic area (mean = 1.34), weak urinary stream (mean = 1.33), and perineal bulging or swelling (mean = 1.17). Overall, the incidence of symptoms was generally low-to-moderate, with most respondents reporting no discomfort or only mild difficulty (Table 3).

### 3.4. Group Differences in PFM-Related Symptoms

Age and marital status were the areas in which a large range of differences could be seen. Greater levels of symptoms were reported by participants over the age of 55 (compared with younger and middle-aged groups), with post hoc analysis suggesting that this age group was much more affected than their younger counterparts (*p* < 0.001). Whether the respondents were married or not also had a link with the degree of experienced symptoms: marital status was associated with symptom burden; however, the divorced subgroup was very small (*n* = 3), limiting reliable post hoc comparisons. Overall, older age was associated with higher symptom burden, and marital status showed group differences; however, estimates for divorced men should be interpreted cautiously due to the very small subgroup (*n* = 3) (Table 4).

### 3.5. Correlation and Regression Analysis

The Spearman correlation analysis highlighted significant associations between PFM-related symptoms and several variables; the most prominent of these were age (r = 0.25, *p* < 0.001), marital status (r = 0.23, *p* < 0.001), smoking (r = 0.13, *p* < 0.01, PFMT), knowledge (r = 0.12, *p* < 0.05), urinary incontinence frequency (r = 0.22, *p* < 0.001), leakage amount (r = 0.20, *p* < 0.001), impact of incontinence on daily life (r = 0.17, *p* < 0.001), and frequent urination as a coping strategy (r = 0.26, *p* < 0.001). Although statistically significant, correlations were small (r = 0.12–0.26), indicating modest associations that are more informative at the population level than for individual prediction.

Two independent associations with PFM-related symptom presence in the adjusted binary logistic regression were current smoking (OR = 1.34, 95% CI: 1.03–1.75, *p* = 0.029) and frequent urination to prevent leakage (OR = 1.54, 95% CI: 1.17–2.04, *p* = 0.002). Given the cross-sectional design, these should be interpreted as associations rather than temporal predictors, and reverse causation is possible (particularly for preventive voiding). Lifestyle and sociodemographic variables, such as place of residence, occupation, and education level, were not independently associated with symptom presence after adjustment (Table 5).

## 4. Discussion

A validated Arabic questionnaire, compiled from the PFDI-20, PFIQ-7, and the ICIQ-UI SF, was used in this analytical cross-sectional survey of Saudi Arabian men (*n* = 458) to estimate the overall burden of PFM-related symptoms and to investigate associated factors. On average, the symptom burden in our sample ranged between low and moderate. Two factors were independently associated with symptom presence in multivariable models: current smoking (OR = 1.34, 95% CI: 1.03–1.75, *p* = 0.029) and regular “preventive” urination to avert leakage (OR = 1.54, 95% CI: 1.17–2.04, *p* = 0.002). No additional sociodemographic or lifestyle variables retained statistical significance after adjustment. However, given the cross-sectional design, these findings should be interpreted as associations and do not establish temporal direction or causality [13]. Accordingly, smoking and preventive voiding are best viewed as potentially modifiable correlates that may help identify men who could benefit from early screening, education, and conservative self-management advice. Prospective and interventional studies are needed to determine whether modifying these behaviors reduces symptom burden.

In this community sample, 86% of men reported no urinary leakage; such episodes as did occur amongst the others were irregular (≈9% at ≤weekly) and only to a small degree (13.1%). A total of 89.1% of respondents reported that there was a negligible effect on their daily lives. Accordingly, the clinical implication is not a universal or intensive pathway but a proportionate, low-intensity screening/education approach targeted to the smaller symptomatic subgroup and focused on conservative self-management where appropriate in symptomatic men. The highest mean scores among the five-item PFM-related symptoms set were for trouble when defecating (1.40) and inability to completely empty (1.36); the next most-reported symptom was heaviness, or pressure, in the pelvic area (1.34), followed by a weak stream (1.33); and finally a perineal bulge, which was the least, at 1.17. Among population surveys of a large size, prevalence estimates tend to be higher when broader ICS-based definitions are applied. For example, 64.3% of adults reported at least ≥1 LUTS in the EPIC study, with the most prevalent symptom being named as nocturia, at 48.6%; symptoms involving storage featured more often than voiding or post-micturition [14]. In an EpiLUTS study carried out across the USA, the UK, and Sweden (≥40 y), 72.3% of men declared that they experienced ≥1 LUTS “at least sometimes”, and 47.9% “at least often” [6]. In Sweden, postal data concerning men ≥ 45 y revealed that there was a 9.2% level of urinary incontinence and that medical advice had been sought by only 46% of sufferers [35].

Across regions of Saudi Arabia, in men over 40, 31.7% displayed a moderate-to-severe degree of LUTSs according to the IPSS, with nocturia again presenting as the most common symptom, while regular or incomplete emptying was associated with poorer quality of life [23]. The variations in this cohort are more likely due to differences in age distribution and the assessment instrument rather than any other cause [12,23]. In terms of the bowel, a worldwide meta-analysis conducted recently indicated that adult fecal incontinence in community settings was about 8%, which demonstrates that men are no strangers to bowel-related pelvic symptoms, particularly as they get older [36]. However, caution is required when interpreting cross-study contrasts, considering heterogeneity, ability to recall, and sampling criteria [37]. Overall, the profile of our cohort revealed a relatively small effect on men’s daily lives but nonetheless demonstrated definite urinary and bowel complaints, findings that impel closer examination of contributory determinants. Diabetes may also influence urinary symptom reporting through mechanisms such as polyuria and diabetic bladder dysfunction/neuropathy affecting bladder sensation and emptying. Diabetes has been associated with a higher burden of LUTSs and urinary incontinence in men and may therefore act as a relevant comorbidity and potential confounder in community surveys. Given the high burden of diabetes in Saudi Arabia, routine screening for diabetes when assessing men with urinary/PFM-related symptoms may help guide conservative management and referral pathways [38].

Two factors remained significantly associated with higher PFM-related symptom scores after adjustment: current smoking (OR 1.34, 95% CI 1.03–1.75) and regular “preventive” urination to avert leakage (OR 1.54, 95% CI 1.17–2.04). Given the cross-sectional design, temporality cannot be established and preventive voiding may represent reverse causation (a coping response to early symptoms) rather than an antecedent predictor. The association with smoking aligns with international evidence, where male smokers demonstrate elevated risks of LUTS phenotypes—including urgency/OAB, nocturia, and IPSS-defined symptoms—in both current and former smokers. Similar associations between smoking and LUTSs have been reported across diverse male populations, although effect sizes vary by age distribution and symptom definitions [25,39]. Proposed mechanisms include nicotine-induced urothelial hypoxia/ischemia and impaired barrier repair, which may heighten symptom perception and bladder irritability [39,40]. Accordingly, integrating smoking-cessation counseling into men’s continence pathways may be a pragmatic adjunct given its broader health benefits, while causality for LUTSs cannot be inferred here. In contrast, preventive voiding reflects a leakage-avoidance coping behavior rather than a direct pathophysiological effect. It may be influenced by contextual constraints (e.g., restricted toilet access), reflect leakage-related concern (“just-in-case” voiding), and could also indicate early awareness of LUTSs before overt urinary incontinence becomes apparent. Current continence guidelines emphasize bladder training to counter this habit, extend inter-void intervals, and reframe it as a modifiable cue for conservative management [41]. In our cohort, higher unadjusted scores were observed for age, BMI, and marital status, but these associations diminished after multivariable adjustment, suggesting overlap with behavioral domains rather than independent effects. Collectively, these associations may serve as modifiable signals to inform conservative counselling and risk stratification in clinical practice. Multicollinearity was assessed (VIF/tolerance). Although the maximum VIF was 9.45 (min tolerance = 0.106), indicating borderline collinearity, the model estimates were interpreted cautiously with emphasis on effect sizes and 95% CIs.

Despite modest, often low-impact, incontinence, the findings remain clinically relevant by highlighting modifiable behaviors and major gaps in help-seeking and conservative self-management, supporting an early physiotherapy-led screening and education pathway. A pragmatic pathway for primary care and physiotherapy may be considered, with concise risk screening and a practical framework for bladder training alongside PFMT. In practice, physiotherapy-led care could emphasize PFMT coaching (with technique verification or biofeedback where feasible), bladder training, and targeted behavioral advice (e.g., urge-suppression strategies, fluid/caffeine timing, and bowel regulation). As these interventions were not assessed in the present cross-sectional study, the following is provided as brief guideline-based context rather than a direct inference from these findings. Current guidelines (AUA/SUFU 2024) suggest making behavioral therapies available at an early stage for idiopathic OAB/LUTSs as part of the shared decision-making process, with stepwise escalation when needed [18]. The International Continence Society stated that bladder training should include the elements that deal specifically with the “just-in-case practice” favored by this cohort. It advocates progressively increasing the interval between voids, developing strategies to suppress urgency, and substantially reducing anticipatory voids [41]. Recent evidence and guidelines support training of the bladder as a low-risk first-line approach, while conservative LUTS male patient care has established PFMT as a recognized treatment for male incontinence, such as in post-prostate rehabilitation [42,43]. Primary care screening of smokers and men practicing “just-in-case” voiding provides a pragmatic entry point. Bladder diaries, coached PFMT, and smoking cessation represent modifiable interventions, while complex cases should be escalated to physiotherapy or urology. In Saudi primary care, family physicians can integrate these elements by conducting brief screening during routine visits and providing initial lifestyle advice. If symptoms persist, patients should be referred to physiotherapy, while those presenting with red flags warrant prompt referral to urology.

The results of this study should be interpreted carefully, taking into account the strengths and limitations of the study, which frame the scope of inference and highlight what priorities there may be in future research. As the outcome is based on symptoms that may involve a range of mechanisms—pelvic floor coordination/strength, detrusor activity, outlet factors, bowel function—it is not therefore a diagnostic PFM-related symptom. Notwithstanding this, validated Arabic items derived from PFDI/PFIQ/ICIQ were used, with robust internal consistency (α ≈ 0.86). The findings are clearly presented as associations typical of patient-reported outcomes. Secondly, cross-sectional designs do not permit causal inference or establish temporal direction; conclusions are restricted to associations, with an emphasis on conservative, modifiable behaviors rather than causal claims. In this context, preventive voiding may reflect reverse causation, whereby it is adopted in response to early symptoms. Thirdly, generalizability may be limited due to regional weighting and large tertiary-education components in the sample. Because recruitment relied on non-probability convenience sampling, selection bias cannot be excluded, and the sample may not reflect the broader population of Saudi men. Accordingly, the prevalence estimates and associations observed in this study should be considered exploratory and interpreted with caution regarding external validity; replication using probability-based, nationally representative sampling is recommended. Having said that, the sample size of 458 enabled stable estimates and transparent reporting of the sample profile, while crucial patterns match population ranges. Lastly, residual confounding such as respiratory problems, constipation, or other unmeasured factors may persist, limiting overall explanatory power. Additionally, several established determinants of male urinary symptoms were not comprehensively captured, including prostate-related history (e.g., benign prostatic enlargement or prior prostate interventions), family history, and dietary factors (e.g., fluid/caffeine patterns). Although BMI was examined, residual confounding from these unmeasured clinical and lifestyle variables may remain. Nevertheless, prespecified multivariable adjustments were applied; the directions observed were consistent with biological plausibility and the prior literature. Accordingly, the smoking association may reflect an adjusted association rather than an independent causal effect, and residual confounding by unmeasured clinical factors cannot be excluded.

These findings provide the following insights for future research: First, temporality could be established by following a cohort of community-dwelling men who regularly report PFM-related symptoms and behaviors. Second, physiotherapy-led interventions should be trialed, combining training the bladder, coaching PFMT (with technique verification/biofeedback where possible), habit-based cues for sedentary workers, and structured smoking-cessation support. Third, broader sampling across multiple regions and sociodemographic groups is needed to enhance national generalizability. Fourth, objective assessments that are culturally accepted (such as transperineally or ultrasound-based pelvic floor evaluation) should be incorporated.

## 5. Conclusions

Amongst a community sample of 458 Saudi Arabian men, PFM-related symptoms ranged from low-to-moderate, with burden patterned by modifiable behaviors. Current smoking (OR = 1.34, 95% CI 1.03–1.75) and regular “preventive/just-in-case” urination (OR = 1.54, 95% CI 1.17–2.04) were independently associated with higher symptom levels. Given the cross-sectional design, these findings should be interpreted as associations rather than causal predictors, but they support a proportionate, rehabilitation-informed primary care approach focused on early screening, education, and conservative self-management, while prospective and interventional studies are needed to confirm temporality and establish a physiotherapy-led care pathway.

## Figures and Tables

**Table 1:** Demographic and lifestyle characteristics of the study participants (*n* = 458).

Variable	Category	*n*	%
**Age**	<25 years	77	16.8
	25–34 years	63	13.8
	35–44 years	135	29.5
	45–55 years	130	28.4
	>55 years	53	11.6
**Marital status**	Single	105	22.9
	Married	350	76.4
	Divorced	3	0.7
**Educational level**	Less than high school	9	2.0
	High school	39	8.5
	Diploma	66	14.4
	Bachelor’s	219	47.8
	Postgraduate	125	27.3
**Nature of work**	Office work (prolonged sitting)	193	42.1
	Field work (requiring physical activity)	145	31.7
	Not employed	83	18.1
	Other	37	8.1
**Daily sitting time**	<4 h	90	19.7
	4–6 h	190	41.5
	6–8 h	107	23.4
	>8 h	71	15.5
**Physical activity**	Daily	132	28.8
	Several times/week	160	34.9
	Rarely	149	32.5
	None	17	3.7
**Smoking status**	Never smoked	233	50.9
	Current smoker	130	28.4
	Former smoker	95	20.7
Health conditions *	Spine or back problems	69	13.4
	Obesity	56	10.8
	Hypertension	54	10.4
	Diabetes	52	10.1
	None	286	55.3

* Multiple responses possible for health conditions. Demographic and lifestyle variables were collected in categorical form and are reported as *n* (%); therefore, continuous summaries (mean ± SD or median (IQR)) were not applicable.

**Table 2:** Awareness, knowledge, and practice of pelvic floor muscle training (PFMT) among respondents (*n* = 458).

Variable	Category	*n*	%
**Heard about PFMT**	Yes	289	63.1
	No	138	30.1
	Not sure	31	6.8
**Sources of information**	Social media	194	39.6
	Websites	142	29.0
	Healthcare professionals	84	17.1
	Friends/family	70	14.3
**Knowledge of PFMT**	Know well	60	13.1
	Basic understanding	205	44.8
	Heard only; do not know how to do it	81	17.7
	No knowledge	112	24.5
**Practice of PFMT**	Regularly	53	11.6
	Sometimes	178	38.9
	Never	227	49.6
**Perceived benefits**	Pain relief	215	33.7
	Core muscle strength	210	32.9
	Improved urinary control	90	14.1
	No perceived benefit	124	19.4

**Table 3:** Distribution of pelvic floor muscle symptoms among respondents (*n* = 458). Symptom severity is presented as mean scores (1–4 scale), with higher means indicating greater discomfort; most participants reported no or mild symptoms.

Variable	Category	*n*	%
**Urinary incontinence (frequency)**	Never	394	86.0
	Once a week or less	41	9.0
	≥2 times per week	19	4.1
**Leakage amount**	None	389	84.9
	Small	60	13.1
	Medium/large	9	1.9
**Impact on daily life**	No effect	408	89.1
	Minor effect (very little/little)	33	7.2
	Moderate–severe effect	17	3.7
**Triggers of leakage**	Urgency	34	7.4
	Cough/sneeze/exercise	10	2.1
	Sleeping	4	0.9
	No clear reason	21	4.6
**Coping strategies**	Frequent urination	87	19.0
	None	371	81.0
**Medical consultation**	Never	437	95.4
	Sometimes/often	21	4.6
**Pelvic floor muscle symptoms (means)**	Defecation difficulty/discomfort	—	1.40
	Incomplete bladder emptying	—	1.36
	Heaviness/pressure	—	1.34
	Weak urine stream	—	1.33
	Perineal bulge/swelling	—	1.17

**Table 4:** Group differences in pelvic floor muscle symptoms by age and marital status (*n* = 458). One-way ANOVA with LSD post hoc tests indicated higher symptom scores among older men (>55 years) and divorced men compared with other groups.

Variable	Test	Test Statistic	*p*-Value	Direction of Difference
Age group	One-way ANOVA	F = 6.85	<0.01	>55 years > younger
Marital status	One-way ANOVA	F = 15.60	<0.01	Divorced > married/single
Educational level	One-way ANOVA	F = 0.60	0.66	NS
Nature of work	One-way ANOVA	F = 0.78	0.51	NS

NS: not significant.

**Table 5:** Correlation and binary logistic regression analysis of pelvic floor muscle symptoms among respondents (*n* = 458). Current smoking and frequent preventive urination were significant independent predictors, while other sociodemographic and lifestyle factors were not.

Variable	Correlation (ρ)	*p*-Value	OR (95% CI)	*p*-Value
Age	0.25	<0.01	1.22 (0.95–1.58)	0.12
Marital status	0.23	<0.01	1.57 (0.73–3.35)	0.25
Smoking	0.13	<0.01	**1.34 (1.03–1.75)**	**0.03**
PFMT knowledge	0.12	0.02	1.07 (0.77–1.48)	0.70
Urinary incontinence frequency	0.22	<0.01	1.59 (0.93–2.71)	0.09
Leakage amount	0.20	<0.01	0.76 (0.37–1.57)	0.46
Daily life impact	0.17	<0.01	1.05 (0.77–1.43)	0.76
Frequent urination (coping)	0.26	<0.01	**1.54 (1.17–2.04)**	**0.002**
Seen a doctor	0.14	<0.01	1.66 (0.75–3.68)	0.21
Education, work, residence	NS	—	NS	—

Abbreviations: PFMT, pelvic floor muscle training; ρ, Spearman’s rank correlation coefficient; OR, odds ratio; CI, confidence interval; NS, not significant. ORs are presented with 95% CIs. A two-tailed *p* < 0.05 was considered statistically significant. Model fit (logistic regression): −2 Log Likelihood = 566.256; Cox & Snell R^2^ = 0.124; Nagelkerke R^2^ = 0.167. Model diagnostics: Hosmer–Lemeshow χ^2^(8) = 16.17, *p* = 0.040; AUC = 0.716; max VIF = 9.45 (min tolerance = 0.106; borderline collinearity—estimates interpreted cautiously). Bold indicates statistically significant results (*p* < 0.05).

## Data Availability

The data presented in this study are available on reasonable request from the corresponding author. The data are not publicly available due to privacy and ethical restrictions.

## Abbreviations

The following abbreviations are used in this manuscript:

ANOVA	Analysis of variance
AUA	American Urological Association
AUA/SUFU	American Urological Association/Society of Urodynamics, Female Pelvic Medicine & Urogenital Reconstruction
BMI	Body mass index
CI	Confidence interval
EAU	European Association of Urology
EPIC	Epidemiology of Incontinence and other Lower Urinary Tract Symptoms (study)
EpiLUTS	Epidemiology of Lower Urinary Tract Symptoms (study)
GATS	Global Adult Tobacco Survey
IBM	International Business Machines (SPSS)
ICIQ-UI SF	International Consultation on Incontinence Questionnaire–Urinary Incontinence Short Form
ICS	International Continence Society
IPSS	International Prostate Symptom Score
IQR	Interquartile range
LUTSs	Lower urinary tract symptoms
NMQ	Nordic Musculoskeletal Questionnaire
OAB	Overactive bladder
OR	Odds ratio
PFDI-20	Pelvic Floor Distress Inventory–Short Form 20
PFIQ-7	Pelvic Floor Impact Questionnaire–Short Form 7
PFM	Pelvic floor muscle(s)
PFMT	Pelvic floor muscle training
SD	Standard deviation
SPSS	Statistical Package for the Social Sciences
STROBE	Strengthening the Reporting of Observational Studies in Epidemiology
SUFU	Society of Urodynamics, Female Pelvic Medicine & Urogenital Reconstruction
VIF	Variance inflation factor
WHO	World Health Organization
